# Supplementary figures and images for: Medermycin Inhibits TNFα-Promoted Inflammatory Reaction in Human Synovial Fibroblasts
Source: Int J Mol Sci. 2023 Sep 8;24(18):13871. doi: 10.3390/ijms241813871 (PMC10531480; doi:10.3390/ijms241813871)

A.

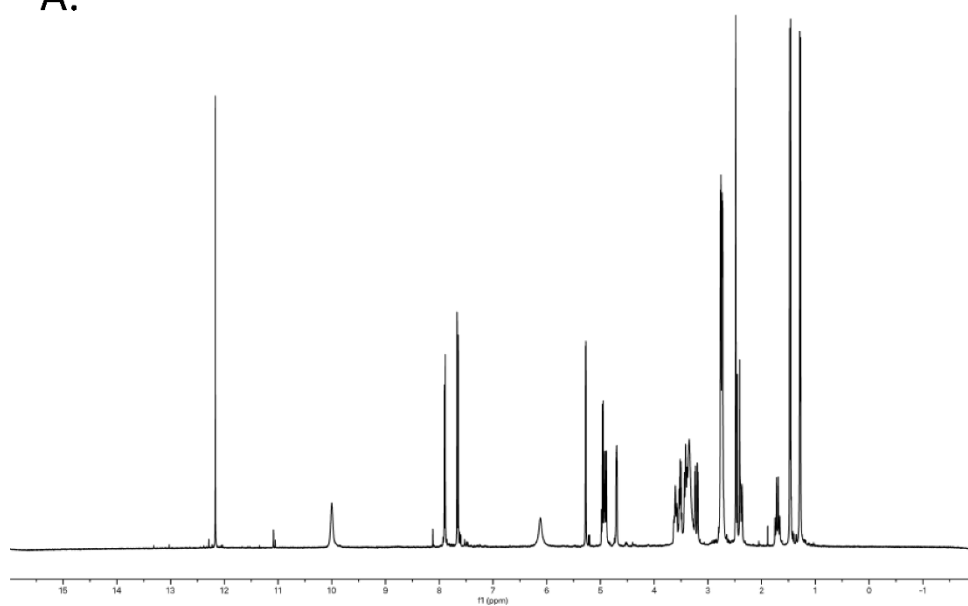

B.

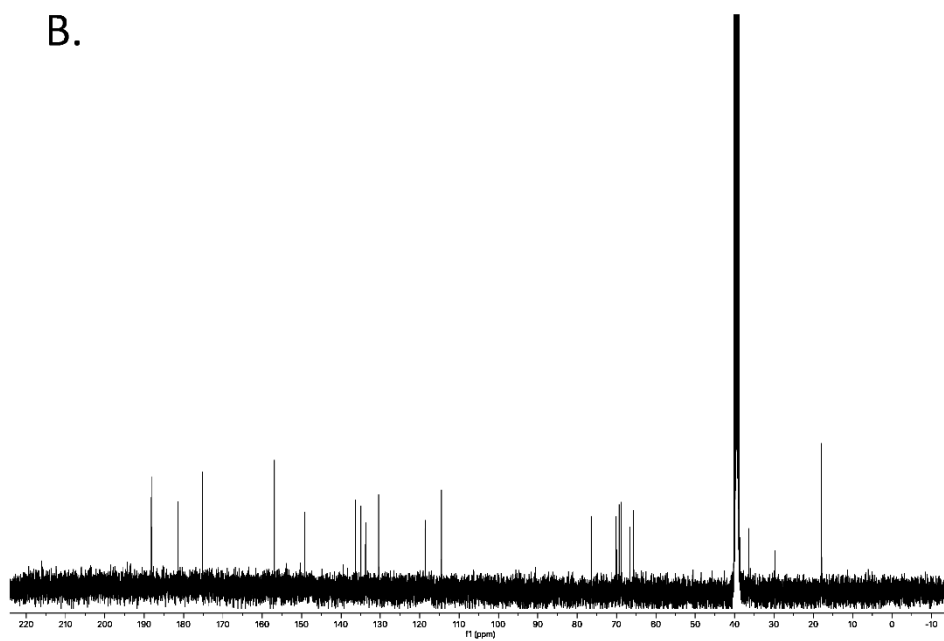

Supplementary Figure S1. <sup>1</sup>H-NMR (A) and <sup>13</sup>C-NMR (B) spectrum of MED in DMSO-*d*<sub>6</sub>

Supplement: Supplementary file 1 [file ijms-24-13871-s001.zip › supplementary figure S1.pdf]
